# Effect of trophectoderm biopsy for PGT-A on live birth rate per embryo in good prognosis patients

**DOI:** 10.1007/s00404-022-06679-x

**Published:** 2022-07-12

**Authors:** Michael S. Awadalla, Ravi Agarwal, Jacqueline R. Ho, Lynda K. McGinnis, Ali Ahmady

**Affiliations:** 1 Institute for Reproductive Health, 3805 Edwards Road Suite 450, Cincinnati, OH 45209 USA; 2 Division of Reproductive Endocrinology and Infertility, Department of Obstetrics and Gynecology, Keck School of Medicine, University of Southern California, Los Angeles, CA USA; 3 Division of Reproductive Endocrinology and Infertility, Department of Obstetrics and Gynecology, Washington University School of Medicine, Washington University in St. Louis, St. Louis, MO USA

**Keywords:** In vitro fertilization, Trophectoderm biopsy, Preimplantation genetic testing, Euploid embryo

## Abstract

**Purpose:**

To determine if blastocyst trophectoderm biopsy for PGT-A is associated with an increased rate of live birth per embryo in good prognosis IVF patients at a single center.

**Methods:**

We performed a retrospective cohort study of good prognosis embryo transfer cycles at a single center from 1/1/2017 to 12/31/2019. We evaluated the rate of live birth per embryo with and without PGT-A for transfer of embryos in two groups of good prognosis patients: embryos from donor oocytes and embryos from autologous oocytes with maternal age less than 35 years at oocyte retrieval. Two-sided Fisher’s exact tests were used for comparisons between groups.

**Results:**

After transfer of embryos created from donor oocytes the live birth rate per euploid embryo was 70.6% (24/34) compared to 34.3% (35/102) for untested embryos for a rate difference of 36.3% (95% CI 18.4–54.1%, *p* < 0.01). After transfer of embryos created from autologous oocytes with maternal age less than 35 years at oocyte retrieval the live birth rate per euploid embryo was 70.0% (49/70) compared to 52.5% (53/101) for untested embryos for a rate difference of 17.5% (95% CI 3.0–32.0%, *p* = 0.03).

**Conclusions:**

In good prognosis patients at our center the live birth rate per euploid blastocyst was higher than for untested blastocysts.

## What does this study add to the clinical work

**Table:** 

In this study we present an analysis of the most recent data on PGT-A for good prognosis patients at a single center. This analysis is unique in that it only examines data after competency in embryo biopsy technique has been achieved by the embryologist.	

## Introduction

Theoretically PGT-A has the potential to benefit patients of all ages. Although the main benefit is to increase the live birth rate per embryo transferred, additional benefits include decreased rate of spontaneous abortion and genetically abnormal pregnancy [1–3]. At our center the euploidy rate ranges from 76% at a maternal age of 26 years (at oocyte retrieval) to 24% at a maternal age of 43 years [4]. Clearly, there is more potential benefit of PGT-A at older ages. However, there is still an expected benefit of PGT-A for younger patients. While PGT-A is not able to improve cumulative success rates from a given batch of embryos, it helps to increase the live birth rate per embryo transferred by avoiding transfer of aneuploid embryos. So is PGT-A beneficial in good prognosis patients such as those using donor oocytes or autologous oocytes with a maternal age less than 35 years? Current published literature shows conflicting results.

Several studies have shown no benefit. A large retrospective paired cohort study of donor oocyte–recipient cycles found no difference in live birth comparing paired outcomes after PGT-A versus no PGT-A when evaluating outcomes of the first or all embryo transfers from 6 frozen donor oocytes [5]. A 2017 SART CORS database study found a reduced odds of live birth per cycle with PGT in donor–oocyte recipient cycles from 2005 to 2013 [6]. One study did not show an increased rate of ongoing pregnancy in a retrospective analysis of donor oocyte single embryo transfers with PGT-A compared to without PGT-A from 2011 to 2016 [7]. A retrospective single center study did not show any difference in live birth rate for single blastocyst transfers in women less than or equal to 37 years when comparing transfer of PGT-A tested embryos to untested embryos [8].

On the other hand, some studies have shown increased ongoing pregnancy rates after PGT-A compared to morphological selection only. A 2012 prospective randomized study by Yang et al. in patients under 35 years found an ongoing pregnancy rate after fresh blastocyst single embryo transfer of 69% using selection with PGT-A by aCGH compared to 42% with selection by morphology alone [9]. A recent large multicenter randomized clinical trial found no difference in ongoing pregnancy rate between selection by PGT-A or morphology overall but did find a 14% increase in ongoing pregnancy rate with PGT-A in a post hoc analysis of women aged 35–40 years [10].

There are two main reasons we would not want to use PGT-A: misclassification of viable embryos as aneuploid and damage to the embryo from the biopsy [11]. A recent large nonelection study found 0 out of 102 single aneuploid embryos progressed to live birth [1]. This indicates that at some centers misclassification is likely very low. Embryo damage from the biopsy technique could theoretically result in loss of implantations or adverse obstetrical outcomes. Currently data seems to suggest that loss of implantations from the biopsy may be high at some centers and negligible at others. This may account for different outcomes reported from studies performed at different centers. We have found that loss of implantations from embryo biopsy likely decreases with increasing embryologist experience with embryo biopsy over the first few years of performing the technique [12]. Although there does not seem to be any clinically significant adverse obstetrical outcomes after embryo biopsy [13], one study found embryo biopsy for PGT to be associated with a small increase in preterm birth with an adjusted odds ratio of 1.20 [14].

The objective of this study was to determine if the live birth rate per embryo at our center is higher for embryos selected by PGT-A than for untested embryos in good prognosis patients.

## Materials and methods

### Study design and patient population

**Fig. 1:**
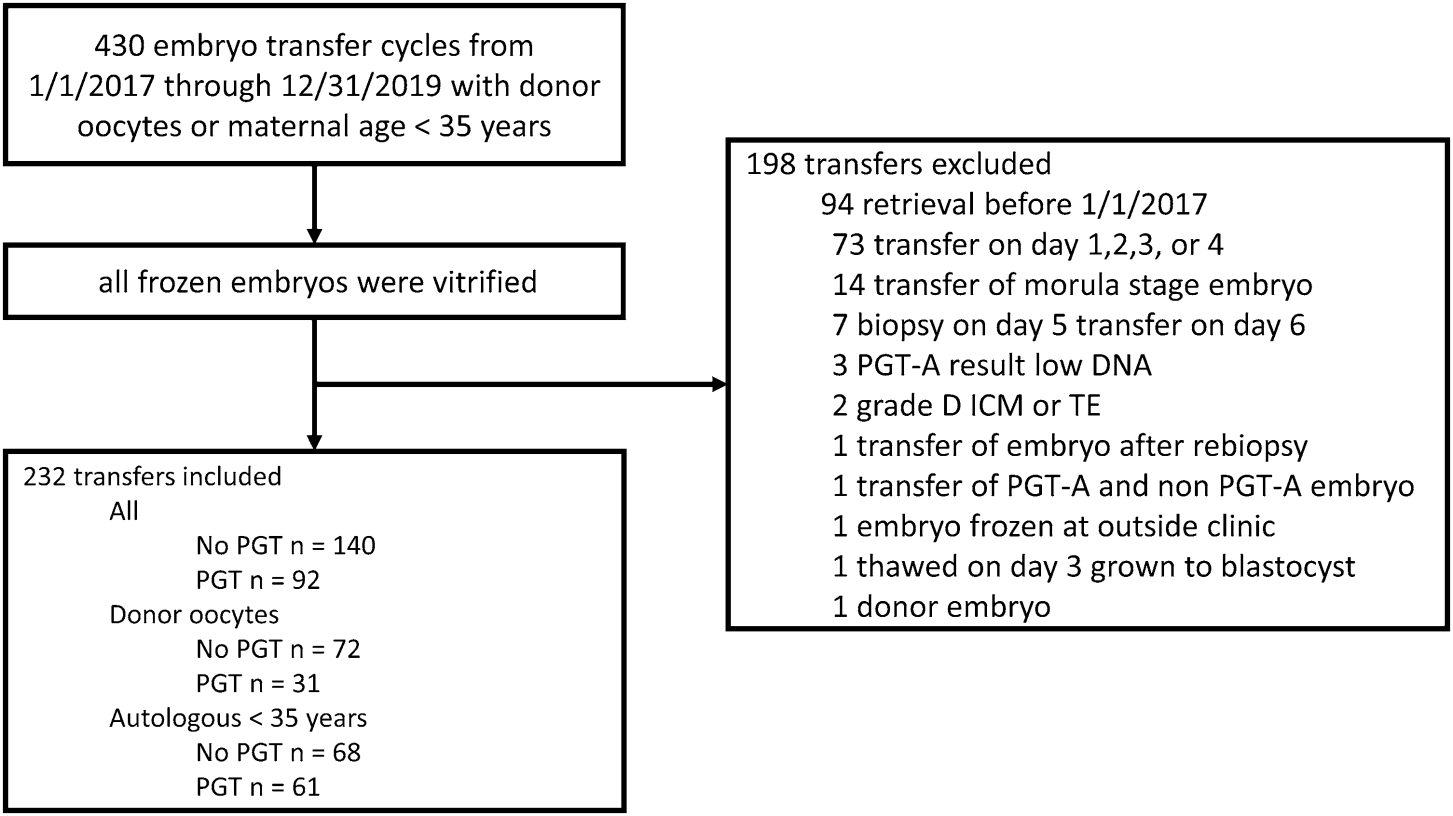
Flow diagram with inclusion and exclusion criteria

This retrospective cohort study included 232 embryo transfers of 307 embryos from 2017 through 2019 at a single center (Fig. 1). We only analyzed our most recent data from 2017 and beyond because our clinic has seen increasing rates of fetal heartbeat per euploid embryo from 2015 when our clinic began performing embryo biopsy through 2017 [12]. Since this increased performance is attributed to a possible increase in proficiency of embryo biopsy, we aimed to analyze our most recent data as this represents our current state of practice.

We performed two comparisons of live birth rate per embryo for embryos from donor oocytes and embryos from autologous oocytes with maternal age less than 35 years at the time of oocyte retrieval. For each of the two comparisons the cohorts were divided by embryo biopsy for PGT-A versus no PGT-A testing (Table 1). In this per embryo analysis the numerator is the total number of live births and the denominator is the total numbers of embryos transferred. The average age of oocyte donors at our center is 28 years [4]. Embryo transfers were excluded if a morula was transferred, embryo was biopsied on day 5 and transferred on day 6, PGT-A result showed low DNA, an embryo was given a D grade for trophectoderm (TE) or inner cell mass (ICM), an embryo was rebiopsied, concurrent transfer of a PGT tested and untested embryo, embryo frozen at an outside clinic, embryo thawed on day 3 and grown to the blastocyst stage, or use of a donor embryo. There were no mosaic embryo transfers. The mean maternal age was 44 years for those using donor oocytes and 32 years for those using autologous oocytes. The mean overall BMI was 24 kg/m^2^ (Table 1). This study was approved by the University of Southern California IRB (HS-21–00,206).

**Table 1:** Baseline demographic and clinical characteristics per transfer

	All		Donor Oocytes		Autologous < 35 years	
	No PGT-A *n* = 140	PGT-A *n* = 92	No PGT-A *n* = 72	PGT-A *n* = 31	No PGT-A *n* = 68	PGT-A *n* = 61
Maternal age at retrieval (years)					32.0 (2.6)	32.2 (2.4)
Maternal age at transfer (years)	38.4 (7.6)	36.3 (6.5)	44.3 (5.8)	43.8 (5.6)	32.2 (2.7)	32.5 (2.3)
BMI (kg/m^2^)^a^	24.6 (4.4)	24.1 (6.3)	23.3 (2.5)	22.8 (6.9)	25.8 (5.3)	24.7 (5.9)
Endometrial thickness (mm)	9.2 (1.6)	9.5 (1.8)	9.0 (1.5)	9.4 (1.9)	9.5 (1.5)	9.5 (1.7)
Number of embryos transferred	1.45	1.13	1.42	1.10	1.49	1.15
Frozen oocyte	18 (13%)	2 (2%)	17 (24%)	2 (6%)	1 (1%)	0
Frozen embryo	83 (59%)	92 (100%)	35 (49%)	31 (100%)	48 (71%)	61 (100%)
Gestational Carrier	9 (6%)	11 (12%)	6 (8%)	7 (23%)	3 (4%)	4 (7%)
Race/ethnicity						
White	70 (50%)	53 (58%)	32 (44%)	22 (71%)	38 (56%)	31 (51%)
Asian	29 (21%)	25 (27%)	18 (25%)	5 (16%)	11 (16%)	20 (33%)
Hispanic	26 (19%)	7 (8%)	14 (19%)	3 (10%)	12 (18%)	4 (7%)
African American	9 (6%)	1 (1%)	4 (6%)	1 (3%)	5 (7%)	0
Multiple	6 (4%)	5 (5%)	4 (6%)	0	2 (3%)	5 (8%)
Unknown	0	1 (1%)	0	0	0	1 (2%)
PGT Indication						
Aneuploidy screening		73 (79%)		26 (84%)		47 (77%)
Advanced age		3 (3%)		2 (6%)		1 (2%)
PGT-M/SR		3 (3%)		0		3 (5%)
Sex determination		3 (3%)		0		3 (5%)
Desired SET		8 (9%)		3 (10%)		5 (8%)
Recurrent IVF Failure		1 (1%)		0		1 (2%)
HLA determination		1 (1%)		0		1 (2%)

Data are given as mean, mean (SD) or *n* (%). *PGT* preimplantation genetic testing, *M* monogenic/single gene defect, *SR* structural rearrangements, *RPL* recurrent pregnancy loss, *SET* single embryo transfer, *IVF* in vitro fertilization, HLA human leukocyte antigen. ^a^*BMI* available for 91% of cycles

### IVF protocols

Our IVF protocols have been described previously [13, 15]. Briefly, gonadotropin-releasing hormone (GnRH) antagonist, GnRH agonist suppression, and GnRH flare suppression stimulation protocols were used. A modified version of the Gardner and Schoolcraft blastocyst grading system[16] was used where occasionally a letter grade of D is assigned for very poor quality ICM or TE. Blastocyst biopsy and vitrification was performed once an embryo had a blastocoel greater than half of the volume of the embryo (expansion stage 2 or greater). When embryo biopsy was not performed embryos were vitrified once they reached expansion stage 1 (blastocoel less than half the volume of the embryo) or greater. Embryo culture was carried out until day 7 at which time embryos were transferred, cryopreserved, or discarded.

Blastocyst biopsy was performed using a Lykos laser (Hamilton Thorne, Beverly, MA, USA) to separate the biopsy cells from the embryo. 90% of embryo biopsies were performed by the laboratory director and 10% were performed by a senior embryologist. PGT-A was performed using next generation sequencing (Progenesis, La Jolla, CA, USA). 95% of frozen embryo transfers at our center were performed in programmed cycles using 50 mg of IM progesterone in ethyl oleate daily and 200 mg of micronized progesterone vaginally twice a day. Blastocyst transfer was performed on day 6 of progesterone, approximately 108 h after the start of progesterone exposure.

### Statistical analysis

Fisher’s exact test was used to compare rates of live birth per embryo (Stata version 16.1, StataCorp, College Station, TX, USA). This study had 80% power to detect a 17% absolute difference in live birth rate per embryo between the PGT-A and non PGT-A groups for all data combined at a two-sided alpha level of 0.05. For embryos from donor oocytes there was 80% power to detect a 28% difference at this same significance level. For embryos from autologous oocytes there was 80% power to detect a 22% difference at this same significance level. There were no clinically significant differences in baseline demographics or clinical characteristics that were expected to have a large impact on live birth rates (Table 1). Embryo quality based on morphology and day of embryo blastulation was slightly worse in the PGT-A cohort for embryos from donor oocytes (Table 2). Worse morphology is an expected association with PGT-A since PGT-A selects first for euploidy and second for embryo morphology. For this reason, statistical comparison without adjusting for embryo morphology was performed as this most closely corresponds to clinical decision making.

**Table 2:** Distribution of embryo morphology and day of biopsy

	All *n* = 307		Donor Oocytes *n* = 136		Autologous < 35 years *n* = 171	
	No PGT-A *n* = 203	PGT-A *n* = 104	No PGT-A *n* = 102	PGT-A *n* = 34	No PGT-A *n* = 101	PGT-A *n* = 70
Day 5 Good (AA/AB/BA)	49 (24%)	22 (21%)	23 (23%)	6 (18%)	26 (26%)	16 (23%)
Day 5 Fair (BB/CB/AC/CA)	96 (47%)	36 (35%)	51 (50%)	9 (26%)	45 (45%)	27 (39%)
Day 5 Poor (BC/CC)	40 (20%)	15 (14%)	24 (24%)	7 (21%)	16 (16%)	8 (11%)
Day 6 Good (AA/AB/BA)	0	1 (1%)	0	0	0	1 (1%)
Day 6 Fair (BB/CB/AC/CA)	9 (4%)	8 (8%)	1 (1%)	6 (18%)	8 (8%)	2 (3%)
Day 6 Poor (BC/CC)	9 (4%)	18 (17%)	3 (3%)	6 (18%)	6 (6%)	12 (17%)
Day 7 Good (AA/AB/BA)	0	0	0	0	0	0
Day 7 Fair (BB/CB/AC/CA)	0	0	0	0	0	0
Day 7 Poor (BC/CC)	0	4 (4%)	0	0	0	4 (6%)

Data are given as *n* (%). *P* < 0.01 for comparison of distributions between PGT-A and no PGT-A for all and donor oocyte comparisons. *P* = 0.18 for comparison of distributions for autologous embryos (Chi-square test, day 6 and day 7 embryos analyzed together as one group)

## Results

After transfer of embryos created from donor oocytes the live birth rate per euploid embryo was 70.6% (24/34) compared to 34.3% (35/102) for untested embryos for a rate difference of 36.3% (95% CI 18.4–54.1%, *p* < 0.01). For untested embryos created from donor oocytes the live birth rate was 30.4% (7/23) when vitrified oocytes were used and 35.4% (28/79) when fresh oocytes were used.

After transfer of embryos created from autologous oocytes with maternal age less than 35 years at oocyte retrieval the live birth rate per euploid embryo was 70.0% (49/70) compared to 52.5% (53/101) for untested embryos for a rate difference of 17.5% (95% CI 3.0–32.0%, *p* = 0.03).

**Fig. 2:**
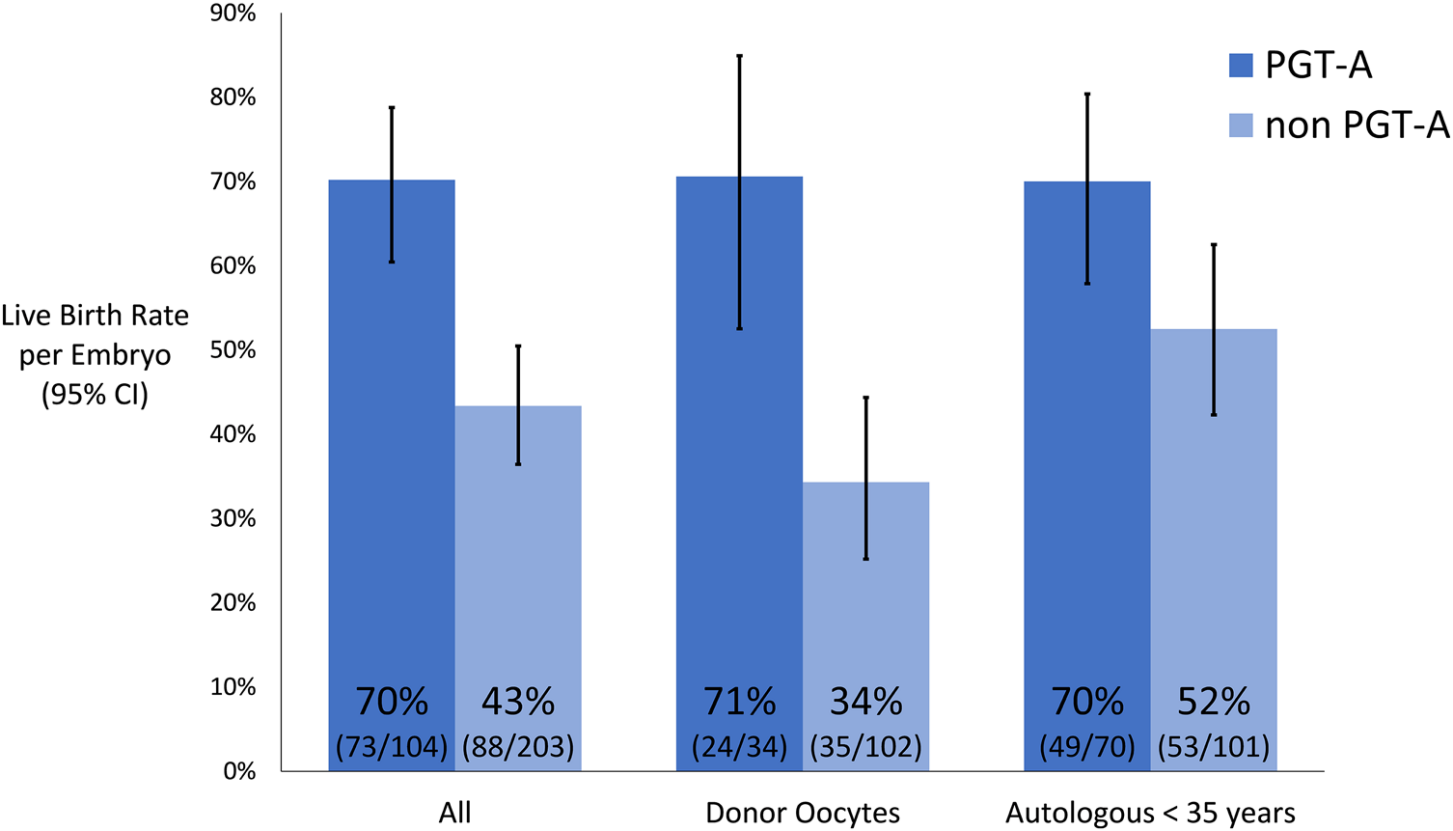
Live birth rate per embryo with 95% CIs. *P* < 0.01 for comparison of live birth rate per embryo for embryos from donor oocytes. *P* = 0.03 for embryos from autologous oocytes. *P* < 0.01 for overall analysis with data from both groups combined

Overall analysis with data from both groups combined showed the live birth rate per euploid embryo was 70.2% (73/104) compared to 43.3% (88/203) for untested embryos for a rate difference of 26.8% (95% CI 15.7–38.0%, *p* < 0.01), as shown in Fig. 2.

## Discussion

There are multiple factors that need to be considered when analyzing euploid embryo transfer data and determining when PGT-A is clinically beneficial. One consideration is if PGT-A correctly identifies which embryos will progress to live birth. Although most PGT-A platforms have not performed clinical validation studies, clinical data from one center suggests that aneuploid embryos rarely progress to live birth [1]. Another consideration is if embryo biopsy causes a loss of implantations or live births. One group of investigators found that biopsy protocols can affect live birth rates [17]. It is likely that different biopsy protocols and embryologist experience contribute to variable rates of loss of implantations between clinics. At present, loss of implantations from embryo biopsy seems to be clinically insignificant at some centers and significant at others. This makes it challenging to interpret data from multicenter studies [10]. Centers with significant loss of implantations from embryo biopsy may be less likely to publish their data than other centers where the PGT-A data look more favorable.

If there is some loss of implantations with embryo biopsy then we would anticipate a smaller than expected increase in ongoing pregnancy for embryos that are euploid by PGT-A testing. In good prognosis patients with high euploid rates, the benefit of selecting euploid embryos may be negated by loss of implantations from the biopsy procedure. However, if there is minimal loss of implantations from the biopsy procedure, we would anticipate increased ongoing pregnancy and live birth rates from euploid embryos at all ages. Looking at our overall analysis we found a 43% live birth rate for untested embryos. With PGT-A and no loss of implantations from the biopsy procedure we would expect this live birth rate to increase proportionally to the euploidy rate. Based on age, approximately 70% of the untested embryos in this study are expected to be euploid [4]. We expect a live birth rate of 0.43/0.70 or 61% for PGT-A tested embryos. Indeed 61% falls within our 95% CI for live birth rate per embryo for PGT-A tested embryos (Fig. 2). Embryo mosaicism factors somewhat into the equation but since less than 2% of embryos are reported as mosaic by our testing platform this has minimal impact on calculating the expected increase in live birth rate with PGT-A [4].

**Fig. 3:**
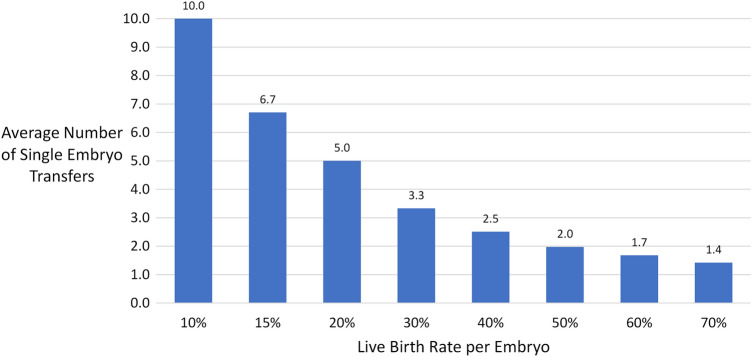
Average number of single embryo transfers needed to achieve one live birth based on live birth rate per embryo

Based on these results, PGT-A is a cost-effective approach for our patients. Going from a 40% to a 60% live birth rate per embryo would decrease the average number of single embryo transfers needed to achieve a live birth from 2.5 to 1.7 transfers, as shown in Fig. 3. At a cost of $4000 per embryo transfer that would be a $3200 savings on average per live birth. The current cost for embryo biopsy and PGT-A is approximately equal to the cost of one frozen embryo transfer cycle. Many good prognosis patients have more than 1 live birth per retrieval and they would have greater cost savings. Based on the current costs of embryo biopsy and PGT-A, the procedure seems approximately cost neutral at worst and cost effective at best for patients who have enough embryos for more than 1 live birth. Cost effectiveness studies based on data more than 5 or 10 years or data from multiple centers may not be applicable to current practice at centers proficient in embryo biopsy and PGT-A [18].

There are some other considerations when deciding if PGT-A is a good option for a specific patient. Insurance may not cover PGT-A in younger patients due to lack of published literature supporting PGT-A use at younger ages. This may change with more clinical experience and more data being published each year. Although live birth rate per embryo is currently the main clinical consideration, euploid embryo transfer is also associated with decreased rates of spontaneous abortion and genetically abnormal pregnancy [1–3]. While PGT-A is able to improve embryo selection among available embryos, it is not able to improve the quality of an individual embryo. The increased live birth rate per embryo is attributed to selecting the best embryo for transfer.

### Limitations

Couples at our center who purchase 6 vitrified donor oocytes for a frozen oocyte embryo transfer cycle typically transfer 1 embryo fresh without PGT-A and cryopreserve any additional embryos. On the other hand, couples using fresh donor oocytes are more likely have PGT-A testing performed, since there are often excess embryos expected and this testing helps select embryos for transfer. With fresh donor oocyte cycles, typically there are 10–20 oocytes retrieved which in general is expected to result in higher quality embryos than cycles starting with only 6 frozen donor oocytes. There may be some uncorrected confounding between PGT-A and use of fresh oocytes. Despite this, transfer of embryos from donor oocytes using PGT-A actually had worse morphology than embryos from donor oocytes not using PGT-A due to the deselection of morphology (giving priority to euploidy rather than morphology) that occurs with PGT-A testing and culture of embryos to expansion stage 2 before embryo biopsy (Table 2). Regardless, prospective studies are needed from centers proficient in embryo biopsy and PGT-A to verify our retrospective data.

In this analysis we included use of embryos from fresh and frozen oocytes, fresh and frozen embryo transfer, transfers to gestational carriers, and single and double embryo transfers. Inclusion of these diverse types of transfers makes the data broadly applicable. However, this analysis is less controlled than ideal. Decisions about proceeding with PGT-A and the number of embryos to transfer are made by the patient and physician after considering all aspects of the patient’s medical history including age, expected number of embryos, number of prior spontaneous abortions, and obstetrical history. Limiting the analysis to single frozen embryo transfers of embryos created from fresh oocytes would add more control but based on our data set the numbers would have been too small to have sufficient statistical power. In the analysis in Fig. 2, the numerator is the total number of live births and the denominator is the total numbers of embryos transferred. We are essentially assuming that each embryo in a double embryo transfer implants independently of the other. This assumption is reasonable since most major endometrial factors are detected with modern ultrasound monitoring and uterine cavity imaging. We routinely performed saline infusion sonograms on all patients prior to embryo transfer. At least one study supports this conventional thinking that embryos implant independently of other embryos transferred concurrently [19].

## Conclusions

PGT-A of embryos from good prognosis patients likely increases the live birth rate per embryo transferred if loss of embryo implantations from the biopsy is low. These results support the use of PGT-A in good prognosis patients at centers with data to support proficiency in this technique.
